# Randomized trial evaluating the effectiveness of within versus across-category front-of-package lower-calorie labelling on food demand

**DOI:** 10.1186/s12889-020-8434-1

**Published:** 2020-03-12

**Authors:** Eric Andrew Finkelstein, Felicia Jia Ler Ang, Brett Doble

**Affiliations:** Programme in Health Services & Systems Research, Duke-NUS Medical School, 8 College Road, Singapore, 169857 Singapore

**Keywords:** Front-of-pack labeling, Nutrition labeling, Food intake, Calories, Online grocery store

## Abstract

**Background:**

Several front-of-pack (FOP) labels identify healthier options by comparing foods within product categories. Alternative approaches label healthier options by comparing across categories. Which approach is superior remains unknown. The objective of this study was to test the effect of a within-category versus across-category FOP lower calorie label on 1) the percentage of labeled products purchased, 2) several measures of calories purchased (total, per dollar and per serving), and 3) total spending. We also tested the moderating effects of hunger and mood on purchasing patterns.

**Methods:**

Using an online grocery store, we conducted a 3 × 3 crossover trial involving actual purchases with 146 participants randomly exposed to: 1) no labeling control; 2) within-category lower calorie labels, and; 3) across-category lower calorie labels. We labeled the 20% of products with the lowest calories per serving within or across categories. Purchases were compared using a fixed effects regression on first-differenced outcomes.

**Results:**

Relative to the control condition, there was a 3 percentage point increase (*p* = 0.01) in labelled products purchased in the within-category arm and a non-significant decrease of 1 percentage point (*p* = 0.711) in the across-category arm. There was no significant difference in the proportion of labeled products purchased between the two labelling conditions. Neither strategy resulted in reductions in any measure of calories purchased or in total spending. When limited to beverages, there was a 398 cal reduction (*p* = 0.01) in the within-category arm and a 438 cal reduction (*p* < 0.01) in the across-category arm versus the control. Mood and hunger did not modify the effects for either strategy.

**Conclusions:**

Results provide evidence that both labelling strategies have the potential to influence food purchasing patterns. However, we cannot definitely state that one labelling approach is superior or even that an increase in the proportion of labelled products purchased will lead to a reduction in calories purchased.

**Trial registration:**

The American Economic Association’s registry for randomized controlled trials, RCT ID: AEARCTR-0002325; Prospectively Registered October 06, 2017. In compliance with ICMJE policy, the trial was also registered on Clinicaltrials.gov, RCT ID: [NCT04165447]. Retrospectively Registered 11 November 2019.

## Background

There has been a global upward trend in obesity rates [1, 2], putting populations at increased risk for weight-related diseases such as type 2 diabetes, heart disease, and several types of cancer [2, 3]. Changes in lifestyle patterns, including a rise in consumption of high-calorie foods and beverages, [1, 4] is partly responsible for this trend. As a result, policy-makers have implemented or are considering strategies aimed at encouraging healthier food consumption [3, 5–8]. One such strategy is increased use of food labels to help consumers identify healthier options.

Many countries either mandate or recommend the inclusion of a nutritional information panel (NIP) on pre-packaged foods and beverages to assist consumers in making healthier food choices [9]. However, the NIP is difficult for many consumers to understand and there is little evidence to suggest that this strategy has positively influenced dietary outcomes [10, 11]. Previous work has also found that consumer usage and comprehension of the NIP is limited and moderated by nutritional literacy [12]. In contrast, some front-of-pack (FOP) labels have been shown to be more salient and simpler to understand [13–15]. As a result, increased attention has been paid to optimizing usage of FOP labels in efforts to improve diet quality.

A variety of strategies have been employed concerning which foods to label. On one hand, some FOP labels, including Singapore’s Healthier Choice Symbol (HCS) label, use the label to indicate that a product (e.g., a biscuit) is healthier based on selected nutritional attributes when compared to other products *within the same product category* (e.g., compared to other baked goods). The within-category strategy of the HCS label is similar to other FOP labels like the Nordic Keyhole in Sweden [16, 17] and the Choices Logo in the Netherlands [18, 19]. Contrarily, the United Kingdom’s Multiple Traffic Lights (MTL) label and other traffic light style labels use a color-coded system to display a products’ relative healthiness when compared to all products, with the only separation being between foods and beverages.

These competing approaches raise the question of which strategy is more likely to positively influence diet quality. If shoppers consider all foods as potential substitutes, then an across-category labeling approach may be preferable because the labels might encourage shoppers to completely avoid some less healthy categories in favor of others. However, if there are certain products that shoppers are inclined to purchase regardless of labels and they all receive the same label (e.g., all labeled as foods to avoid), then the labels may cease to offer any value to consumers. In that case, a within-category approach that identifies the least bad options within the category would be more effective. To date, no study has directly tested these competing approaches using actual purchases. That is the focus of this study.

We test within- versus across-category labeling using a positive FOP *‘Lower Calorie’* label applied to select lower calorie products in an online grocery store. We focus on a positive message promoting lower calories, given that excess caloric intake is a primary cause of obesity and because consumers generally understand that consuming fewer calories is better [20]. We hypothesize that, although both labels will positively influence the proportion of labeled (i.e., increased proportion of lower calorie) products purchased (primary) and reduce calories purchased relative to the control arm, the greatest improvements would occur in the within-category labeling condition. This is because we expect more substitution to occur within, as opposed to across, product categories, especially in the presence of a positive FOP label that identifies the healthier products within the category. We test this hypothesis for all foods and separately for beverages only, recognizing that several jurisdictions have implemented or considered separate FOP labels solely for beverages. We also expect that FOP lower-calorie labels will be less effective for beverages than for foods because consumers are likely to have an easier time identifying lower-calorie beverages in the absence of these labels given that lower-calorie beverages are typically already marked as diet, unsweetened, or zero calorie [21]. Lastly, we test whether results are moderated by being unhappy or hungry at the time of the shop. This is likely as a negative mood has been associated with greater impulsivity and less control [22] and hungry grocery shoppers have been shown to choose energy-dense food more frequently [23], and to find it more rewarding [24]. All hypotheses were pre-specified.

## Methods

### Online grocery store

An online grocery store (NUSMart Online Grocery Store) was developed for testing these hypotheses (https://nusmart.duke-nus.edu.sg). At the time of the trial, NUSMart contained over 3200 food and beverage products commonly purchased at local supermarkets in Singapore. The web store was designed to mirror actual web-based grocery stores in Singapore, such as RedMart Online Supermarket (https://redmart.com) or Fairprice Online (https://fairprice.com.sg), in both look and feel. It contained products across major food & beverage categories, including:

- Beverages
- Dairy products
- Snacks
- Meats & seafood
- Cereals, bakery & spreads

All products included pictures of the items, current retail price and product descriptions. NUSMart operated similar to other on-line grocery stores with a cart that filled as consumers shopped and the ability to add and remove products and review purchases before hitting the checkout button.

### Participants and procedures

There were no face-to-face visits between the study team and participants as all study-related procedures were conducted online. A power calculation revealed that at least 140 participants were required to detect differences between any two arms assuming an effect size of 0.3 (a relatively small effect) or larger, for the proportion of the basket represented by *Lower Calorie* products (primary outcome). The calculation assumed a two-tailed test, three comparisons, power of 0.9, alpha of 0.05, and a crossover design. Based on these inputs and an assumed attrition rate of 20%, our target sample was 168.

Participants were recruited from existing users of the online grocery store, RedMart, via Facebook advertisements. At the point of study, RedMart was the largest online grocery store in Singapore and thus had the largest customer base. Facebook advertisements were chosen as the recruitment platform as it was RedMart’s main platform for online communication with their consumers. Recruiting existing online grocery shoppers ensured that participants would be comfortable with online shopping. Prospective participants were directed from RedMart’s Facebook page to the study website (https://nusmart.duke-nus.edu.sg) and asked to complete an online screening questionnaire to determine their eligibility for the study. Potential participants were eligible only if they were 21 years of age or above, the primary grocery shopper for their household, and a registered RedMart shopper.

Potential participants who were both interested and eligible were then asked to complete: 1) a registration form containing name, delivery address, National Registration Identity Card number, mobile number and email address; 2) an online consent form; and 3) the baseline questionnaire. Upon completion of the registration form, the website created the participant account and unique participant identification number for use throughout the study.

**Fig. 1:**
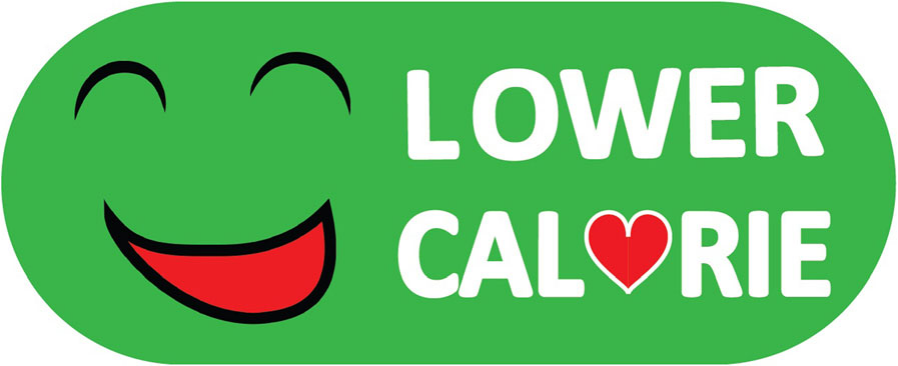
*‘Lower Calorie’* logo used in the LoCal study

We designed a simple *‘Lower Calorie’* directive logo (see Fig. 1) [25] to test the hypotheses. We made it primarily green in color as green labels have been shown to increase perceived healthfulness of foods [26] and included a smiley face nested within the label to act as a signal that the label is referring to a positive choice. The final label was thus both simple and directive for ease of comprehension [25].

**Fig. 2:**
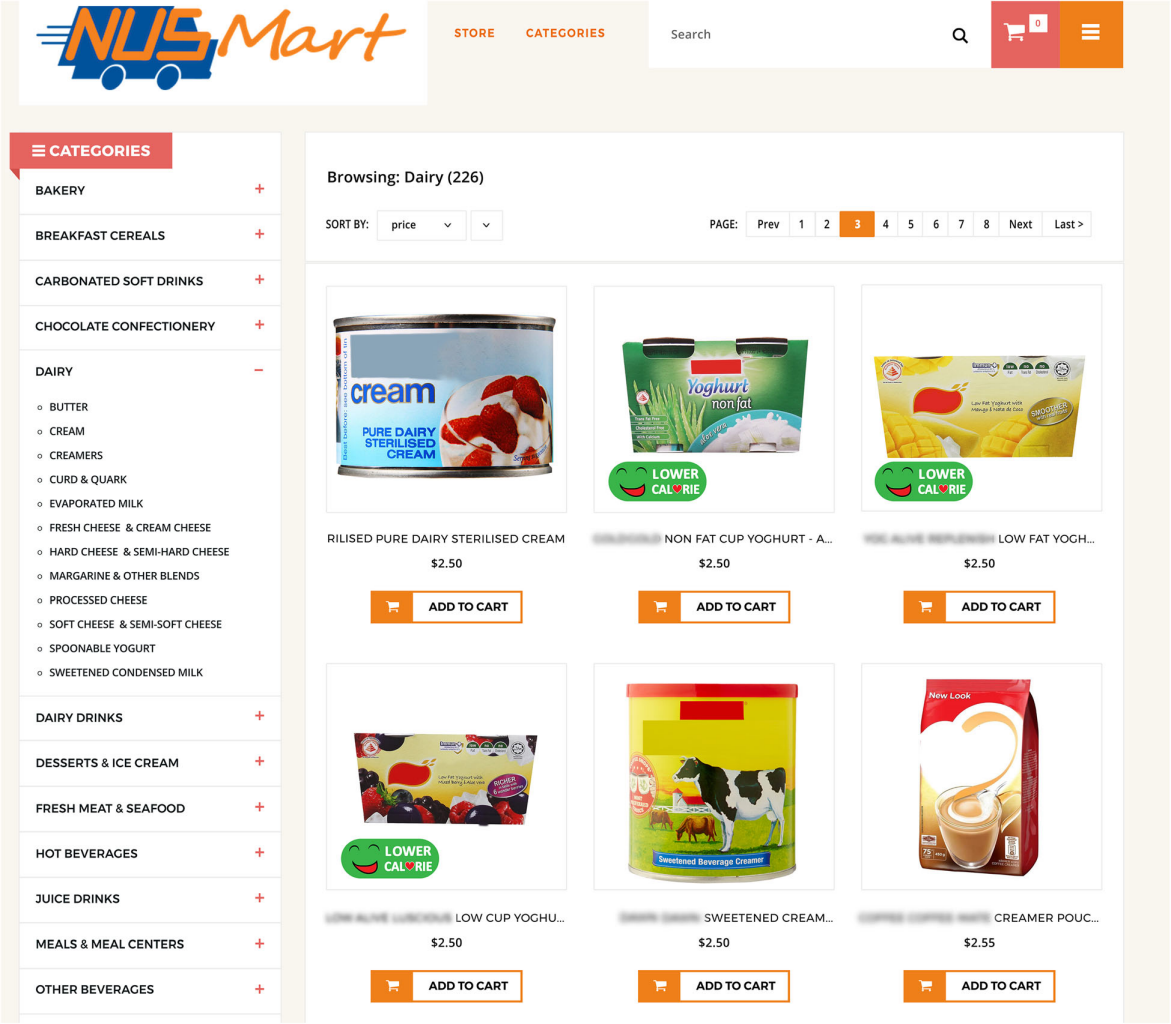
Examples of *‘Lower Calorie’* products as they appeared on NUSMart

Arm 1 was the Control condition, which did not display the label on any products. Arm 2 displayed the label on the 20% of products that were lowest in calories per serving *within each product category* (full category list available in Supplemental Table 1). Arm 3 displayed the label on the 20% of *all* products that were lowest in calories per serving. Prior to conducting the analysis, we standardized the serving size by using the mean serving size within each subcategory. This standardization ensured that similar products were compared equally as serving sizes can be arbitrarily set by the manufacturers [27]. The labels were displayed below the product images (See Fig. 2).

Using a crossover design, all participants were exposed once to the three shopping conditions (1xControl, 1xWithin-category, 1xAcross-category) in random order (see Supplemental Table 2). Each participant was randomly assigned at baseline to 1 of 6 groups that varied in sequence of shopping conditions and which shopping tasks resulted in an actual food delivery. Participants were asked to shop once a week for a total of three weeks and were told that at least one and up to all three of their grocery orders would actually be purchased using their credit card. The result was only revealed to them after they hit the checkout button, which led to their weekly shop being recorded for inclusion in the study. The positive probability of having to purchase and receive the chosen products increased the chance that the purchases were an accurate reflection of participants’ actual shopping patterns. For each shop, there was a minimum spend of SGD50 and a maximum spend of SGD250. A minimum spend ensured that participants completed a typical weekly grocery order. A maximum spend was intended to make the study more manageable. Participants were informed in the consent form before enrollment, and a pop-up message appeared on-screen if they attempted to checkout a cart below or above the minimum and maximum values.

The grocery orders that needed to be fulfilled were ordered and delivered in partnership with RedMart. Following each shopping task, participants completed a brief survey to assess their mood and hunger level. ‘Mood’ took the values 1–5 where 1 was ‘very happy’ and 5 was ‘very unhappy’. ‘Hunger’ took the values 1–10, where 1 was ‘not at all hungry’ and 10 was ‘extremely hungry’. Participants who completed all study elements were rewarded with SGD75 worth of RedMart electronic vouchers.

### Outcome measures

The primary outcome is the proportion of the basket represented by *Lower Calorie* products as this is the most direct test of the influence of the labels. Since it is possible that consumers could respond to the labels by purchasing more or a different distribution of both labeled and unlabeled products and therefore not reduce their net caloric purchases, we also included the following secondary outcomes:

- *Calories purchased per dollar spent (in kcal per $)*
- *Total Spending ($)*
- *Total calories purchased (in kcal)*
- *Calories per serving (in kcal/serving)*

### Statistics

To test our hypotheses, individual-fixed effects regressions using a first difference approach were estimated. Each participant generated two observations, with each dependent variable being the difference in the outcome for each treatment condition (Within-category and Across-category) relative to the Control condition. For secondary outcomes, executing this approach was straightforward. However, in the case of the primary outcome (proportion of labeled products purchased), even though there is only one Control condition, we needed to identify two proportions, one for the products that would have been labeled in the Within-category arm and one for the products that would have been labelled in the Across-category arm. We therefore generated two proportions for each Control shop and subtracted each treatment condition from its corresponding proportion. The regression specification was then estimated as follows:

1 $$ \Delta  Outcome\ relative\ to\ Control=\alpha +{\beta}_A Across+{\epsilon}_i $$

where subscript i = participant and ‘Across’ is a dummy variable equal to one if the order is placed under the Across-category arm and zero otherwise. The following hypotheses were then tested:

*α* > 0 tests whether the outcome is greater in the Within-category arm relative to Control.

*α* + *β*_*A*_ > 0 tests whether the outcome is greater in the Across-category arm relative to Control.

*β*_*A*_ > 0 tests whether the outcome is greater in the Across-category arm relative to the Within-category arm.

We then tested whether the impact of the label is moderated by mood and level of hunger at the time of purchase using the following equation:

2 $$ \Delta  Outcome\ relative\ to\ Control=\alpha +{\beta}_A Across+{\beta}_2 Moderator\ Dummy+{\beta}_3 Moderator\ Dummy\ x\  Across+{\epsilon}_i $$

The ‘Moderator Dummy’ takes on the value = 1 for values of mood and hunger that lie above the median, and 0 for values that lie at or below the median. The median for ‘Mood’ was 2 and the median for ‘Hunger’ was 3. For ease of presentation, we defined above the median as hungry or unhappy. We then tested the following hypotheses:

*β*_2_ < 0 tests whether being hungry or unhappy at the time of purchase will diminish the impact of labeling in Within-category arm relative to Control.

*β*_2_ + *β*_3_ < 0 tests whether being hungry or unhappy at the time of purchase will diminish the impact of labeling in Across-category arm relative to Control.

We conducted the analyses on total baskets and separately for beverages only. For all regressions, standard errors were clustered at the participant level to account for correlation within individuals across shopping tasks. All regressions were run in Stata Version 15.2 (Stata Corp LP, College Station, TX).

## Results

### Sample

**Fig. 3:**
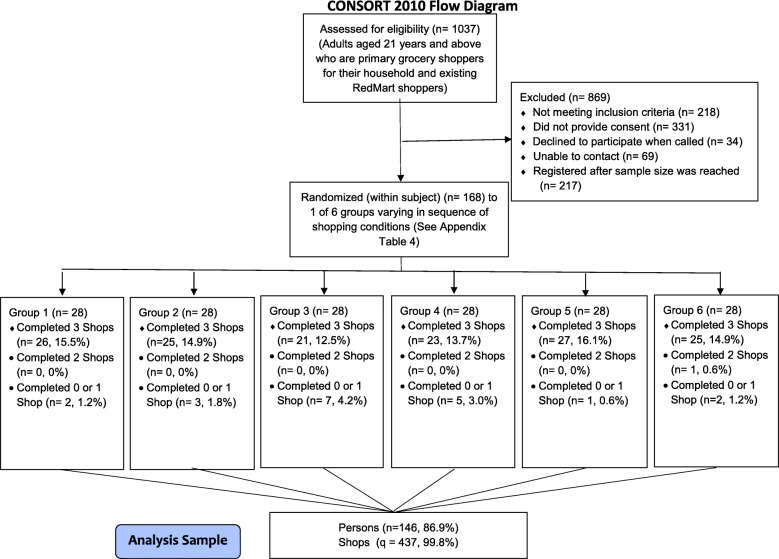
CONSORT Flow Diagram for participant recruitment and randomization

Participant flow for recruitment and randomization is presented in Fig. 3. From October 2017 to April 2018, 168 participants were randomized, 148 began the shopping exercise, one person completed only two shopping tasks and 145 completed all three shopping tasks. This generated an analysis dataset of 437 unique sales orders. The final analyzed sample reflected data from 87% (146 participants) of the recruited sample as 13% (22 participants) of participants were randomized but did not start (*n* = 20) or complete (n = 2) the shopping exercise. Table 1 presents the characteristics of the sample. The sample was largely ethnic Chinese (92.5%) and the mean age was 35.0 years (SD = 5.7). The average body mass index (BMI) was 23.4 kg/m^2^ (SD = 3.6). The majority (78.8%) were female.

**Table 1:** Descriptive statistics (*n* = 146)

Variable	Mean / Proportion of Sample
	Mean / % (S.D.)
Age (years)	35.0 (5.7)
BMI (kg/m^2^)	23.4 (3.6)
Female (%)	78.8
Household size (mean)	3.5 (1.6)
Ethnicity (% Chinese)	92.5
University education and above (%)	84.2
Household income above $10,000 per month (%)	38.4

Table 2 presents the unadjusted values of the primary and secondary outcome variables for the study arms. Regression coefficients for estimating labeling effects are reported in Table 3.

**Table 2:** Unadjusted means of primary and secondary outcome variables for the study arms (*N* = 146)

Outcome	Proportion of (unlabeled) labeled products purchased (i.e., the counterfactual) (%)	Calories per dollar (kcal/$)	Total Spend ($)	Total Calories (kcal)	Calories per Serving (kcal/serving)
Unadjusted Mean of Control Sales Orders^a^	25.0 (vs. Within)	226.0	52.5	11,764.9	155.2
	14.7 (vs. Across)				
95% CI	21.9, 28.0 (vs. Within) 12.1; 17.4 (vs. Across)	199.6; 252.4	51.8; 53.2	10,423.2; 13,106.6	146.2; 164.3
Unadjusted Mean of Within-category Arm Sales Orders	27.9	235.3	52.6	12,265.2	159.7
95% CI	24.5; 31.4	205.1; 265.6	51.9; 53.3	10,720.7; 13,809.8	146.0; 173.3
Unadjusted Mean of Across-category Arm Sales Orders	14.4	238.3	52.6	12,443.2	165.1
95% CI	12.0; 16.7	209.5; 267.1	51.8; 53.5	10,966.1; 13,920.3	151.5; 178.8

^a^Even though there is only one Control condition, we needed to identify two proportions, one for the products that would have been labeled in the Within-category arm and one for the products that would have been labelled in the Across-category arm; CI stands for Confidence Interval

**Table 3:** Regression coefficients for total baskets (*N* = 291)

Outcome	Prop. of labeled products	kCal/Dollar	Total dollars spent	Total kCal	kCal / serving
*α* (Within-category arm relative to Control)	0.03*	9.34	0.11	498.20	3.84
95% CI	0.01; 0.05	−7.37; 26.05	−0.40; 0.63	− 356.95; 1353.35	−1.75; 9.43
*β*_*A*_ (Across-category arm relative to Within-category arm)	−0.03	2.82	0.07	176.21	6.45
95% CI	−0.08; 0.13	− 30.72; 36.36	− 0.96; 1.10	− 1539.99; 1892.41	−4.76; 17.67
*α* + *β*_*A*_ (Across-category arm relative to Control)	− 0.00	12.16	0.18	674.41	10.29
95% CI	−0.03; 0.02	−4.67; 29.00	−0.33; 0.70	− 186.64; 1535.46	4.67; 15.92

* *p* < 0.05; CI stands for Confidence Interval

### Primary outcome (proportion of labeled products purchased)

When focusing on full sales orders (total baskets), as shown in Table 3, the proportion of labelled products purchased was a statistically significant 3 percentage points higher in the Within-category arm compared to Control (*p* = 0.01). The 0.3 percentage point difference between the Across-category arm and Control was not statistically significant (*p* = 0.71), nor was the 2.7 percentage point difference between the Across-category and Within-category arms (*p* = 0.15). When limiting the analysis to beverages only, Table 4 shows that the proportion of labelled products purchased was 4 percentage points higher (*p* = 0.02) in the Across-category arm compared to control, but was smaller and not significantly different when comparing Within-category to Control (*p* = 0.52) or between the Across-category and Within-category arms (*p* = 0.16).

**Table 4:** Regression coefficients for beverages Only (*N* = 184)

Outcome	Prop. of labeled products	kCal/Dollar	Total Dollar Spent	Total kCal	kCal/Serving
*α* (Within-category arm relative to Control)	−0.02	−4.94	−0.68	−398.00*	3.03
95% CI	−0.06; 0.03	−16.02; 6.14	−2.26; 0.91	− 690.91; − 105.10	−3.30; 9.36
*β*_*A*_ (Within-category arm relative to Across-category)	0.06	−9.93	0.90	−40.03	−5.51
95% CI	−0.02; 0.14	−29.71; 9.85	−1.93; 3.73	− 563.28; 483.22	− 16.81; 5.79
*α* + *β*_*A*_ (Across-category arm relative to Control)	0.04*	−14.87*	0.22	−438*	−2.48
95% CI	0.01; 0.08	−23.58; −6.16	−1.02; 1.47	− 668.37; − 207.69	−7.45; 2.50

* *p* < 0.05; CI stands for Confidence Interval

### Secondary outcomes (calories purchased per dollar spent, total calories purchased, total spending, calories per serving)

Table 3 also shows that, for total baskets, none of the 4 secondary outcomes were statistically different across the three arms. For beverages only, Table 4 shows that total calories purchased were 398 lower (*p* = 0.01) in Within-category arm and 438 lower (*p* < 0.01) in Across-category compared to Control. Comparing Across-category and Control, there was also a reduction of 14.87 (*p* < 0.01) calories per dollar spent. However, the remaining differences relative to Control were not statistically significant and there was no significant difference between the two intervention arms for any of the four secondary outcomes.

### Moderator analysis

There were no statistically significant moderating effects for either mood or hunger on the proportion of labeled products purchased under either implementation strategy when analyzed for both total baskets (Tables 5 and 6) and separately for beverage-only purchases (Supplemental Tables 3 and 4). Despite this, and contrary to expectations, being unhappy at the time of purchase led to significantly *lower* calories purchased per dollar spent (108.7 kcal/$, *p* < 0.01), lower total calories (5547.9 kcal, *p* < 0.01) and lower calories per serving purchased (28.9 kcal/serving, *p* < 0.01) in the Within-category arm relative to Control (Table 5). However, these effects were no longer statistically significant when limiting the analysis to only beverages (Supplemental Table 3).

**Table 5:** Regression coefficients for total baskets with moderator mood (*N* = 291)

Outcome	Prop. of labeled products	kCal/Dollar	Total Dollar Spent	Total kCal	kCal / Serving
*α* (Within-category arm relative to control for happy participants)	0.00	69.45*	−0.40	3567.64*	19.81*
95% CI	−0.05; 0.05	27.72; 111.18	−1.69; 0.89	1465.72; 5669.57	6.81; 32.80
*β*_*A*_ (Across-category arm relative to Within-category arm for happy participants)	−0.04	−35.18	0.28	− 1796.14	5.71
95% CI	−0.11; 0.03	− 88.79; 18.43	−1.43; 1.99	− 4618.52; 1026.25	− 12.07; 23.49
*β*_2_ (Unhappy relative to happy participants in Within-category arm)	0.05	−108.65*	0.92	−5547.88*	−28.86*
95% CI	−0.4; 0.15	− 181.10; −36.20	− 1.56; 3.40	− 9203.14; 1892.62	−52.04; − 5.68
*β*_3_ (Unhappy in Across-category arm relative to Within-category arm)	0.02	67.85*	−0.37	3524.29	0.64
95% CI	−0.09; 0.13	−4.35; 140.05	−2.14; 1.40	− 295.13; 7343.70	− 25.19; 26.47
*β*_2_ + *β*_3_ (Unhappy relative to happy participants in Across-category arm)	0.070	−40.800	0.550	− 2023.590	−28.22
95% CI	−0.03; 0.17	− 114.37; 32.76	−2.06; 3.16	− 5744.77; 1697.59	−51.90; − 4.53

* *p* < 0.05; CI stands for Confidence Interval

**Table 6:** Regression coefficients for total baskets with moderator hunger (*N* = 291)

Outcome	Prop. of labeled products	kCal/Dollar	Total Dollar Spent	Total kCal	kCal / Serving
*α* (Within-category arm relative to Control)	−0.01	12.94	0.60	724.36	7.33
95% CI	−0.06; 0.05	−30.38; 56.27	−0.60; 1.81	− 1474.54; 2922.26	−7.16; 21.82
*β*_*A*_ (Across-category arm relative to Within-category arm)	−0.03	4.83	0.33	484.62	5.90
95% CI	−0.11; 0.06	−48.97; 58.63	−1.22; 1.87	− 2241.02; 3210.26	−23.57; 9.05
*β*_2_ (Hungry relative to non-hungry participants in Within-category arm)	0.07	−7.29	−0.99	− 457.03	− 7.05
95% CI	−0.03; 0.17	−88.48; 73.90	−3.62; 1.64	− 4606.39; 3692.33	− 32.84; 18.73
*β*_3_ (Hungry in Across-category arm relative to Within-category arm)	− 0.02	− 3.74	− 0.47	− 591.96	1.28
95% CI	− 0.13; 0.10	−76.97; 69.49	− 2.55; 1.60	− 4339.16; 3155.23	− 24.50; 27.05
*β*_2_ + *β*_3_ (Hungry relative to non-hungry participants in Across-category arm)	0.050	− 11.030	− 1.460	− 1048.99	−5.78
95% CI	−0.04; 0.16	−92.51; 70.46	− 4.11; 1.18	− 5212.06; 3114.08	−31.73; 20.18

* *p* < 0.05; CI stands for Confidence Interval

## Discussion

The primary objective of this study was to determine which of two competing FOP label implementation strategies using *Lower Calorie* labels would have a greater effect on food purchasing patterns and measures of caloric purchases. We hypothesized that individuals would be more likely to make substitutions within categories, rather than across categories and therefore within category labelling would lead to a higher proportion of labeled products purchased. In the total basket analysis, we observed a direction of effect consistent with our hypothesis, but the difference between arms was not statistically significant. For the beverage-only analysis, the direction of effect favored Across-category labeling, although again the difference was not statistically significant.

Our results comparing Within-category to Control in the total baskets analysis and Across-category to Control in the beverage-only analysis shows that even when the label has a statistically significant effect on the proportion of labeled products purchased, it may not have the intended effect on caloric purchases, which in both cases were not statistically different from Control. There are two likely explanations for this. First, consumers may interpret the FOP label as a signal to safely consume the labelled product in greater quantities than they would otherwise [28]. If so, the positive effects of the label could be completely offset through greater purchases of labelled products. Second, consumers may feel entitled to purchase more non-labelled foods as a result of shopping “healthier”. For example, one study conducted in a restaurant found that a successful intervention aimed at promoting healthy entrees also had the unintended effect of increasing purchases of side orders and drinks [29].

Regardless of the cause, evaluating the effectiveness of any FOP label requires more than identifying differences in the percentage of labeled products purchased. As our study reveals, it is possible that labels can simultaneously increase the proportion of labeled products purchased and not improve diet quality.

Contrary to expectations, the labels had a greater impact on reducing beverage calories than food and beverage calories combined, where for the latter they appeared to have no net effect. This may result from a greater consumer focus on limiting beverage calories due to recent efforts to educate consumers about the association between sugar-sweetened beverage intake and obesity and non-communicable diseases (NCDs) [30, 31]. These efforts may have primed consumers to see beverages as a source of calories that should be carefully managed [32, 33], thus enhancing the effectiveness of these labels on beverages.

The discovery of a moderating impact of bad mood in reducing total calories purchased and calories per dollar spent in the Within-category arm runs contrary to prior research, which demonstrated that positive mood tends to increase preferences for healthy foods over indulgent foods [34, 35]. Given these conflicting results and the fact that mood did not moderate the effect of either strategy on the proportion of labelled products, this finding should be viewed with caution.

This study has several strengths. It does not rely on hypothetical purchases that may not reflect actual buying behavior [36]. Recruiting active online grocery shoppers increases external validity, at least for this mode of grocery shopping. The repeated-measures crossover design reduces variability due to unobservable characteristics of individuals as each participant serves as their own control. However, the study is subject to several limitations. First, the NUSMart store at the time this study was conducted had fewer products than would appear in a full grocery store but more products than available in most convenience stores where convenient pre-packaged snacks are more common. It is likely that the number and type of products will influence the effectiveness of FOP labels [37]. Experienced online shoppers may also be less likely to pay attention to labels because they already have established preferences and familiarity with certain products, although this limitation extends to any FOP labeling strategy. Effectiveness of a given label may also wane over repeated shopping trips as consumers try new products and then decide whether or not they want to continue to purchase them. Thus, label use and effectiveness in the real world may be smaller than predicted from our single shop experiment. Although we tested for confounding due to hunger and mood, other visceral factors may also mediate the relationship between labels and food purchases. Finally, we note that food purchases are not synonymous with dietary intake, although purchases likely track intake at the household level. Effectiveness may also be venue-specific and differ for web versus in-store shopping. Given that our experimental store did not offer point-of-sale promotions, attention and label use may be greater than in the real world, which would again suggest real world estimates may be attenuated. Online recruitment also attracts consumers with higher literacy skills and with higher household incomes. Hence, our findings may not be generalizable to shoppers in physical stores in which the characteristics of both the consumers and the environment differ. However, as online grocery shopping is predicted to capture 20% of total grocery retail by 2025 (Food Marketing Institute), our results will be increasingly relevant both within and beyond Singapore. Finally, results could also be influenced by the content, color, size, placement and implementation strategy (e.g., what percent of products are labeled) of the labels and by characteristics of target population, such as degree of health literacy. Testing the independent effect of all of these factors in both controlled research settings and actual retail environments should be areas of future research.

Lastly, it is important to keep in mind that if consumers had perfect information and/or read and understood the nutrition facts panel on the back of products, then FOP labels would not be needed and would have no effect when introduced. However, many real world and experimental studies show that is not the case. As noted in the introduction, FOP labels, ranging from the stop sign to guiding stars have been shown to influence food choice, even when consumers have only a basic understanding of the ratings. In nearly all cases, these labels likely act as a heuristic, allowing for passive shoppers to make a healthier purchase with little effort or understanding of why a product scores better. NuVal exemplifies this in that several studies show it works to influence food choice, yet the algorithm is proprietary; consumers merely know that higher scores mean healthier foods. That appears to be all that is needed to influence their choices.

In our experiment, we did not educate consumers on the underlying logic as to which products received the label. This may have influenced its effectiveness in both labelling conditions. In the real world, any labelling strategy would likely come with an accompanying education effort that explains the label and implementation strategy. Although we expect this educational effort to have only a modest effect on behavior above and beyond the label itself, the greatest effect would likely be for the within-category labelling approach. It is possible that greater education on this approach might lead to a better understanding that these products are not as healthy as the label might suggest, whereas the opposite is true for the across category approach. Testing the incremental effect of education on each implementation strategy should be an area of future research.

## Conclusion

Results indicate that although a within-category Lower Calorie labelling strategy increased purchases of labelled products relative to a no FOP control, there was no significant difference in the effectiveness of the within and across category strategies when applied to all foods and beverages. We also could not find evidence that either strategy is effective at reducing calories purchased when applied to total baskets. Results are more promising when limited to beverages only, as both within- and across-category labels led to a reduction in beverage calories purchased. This suggests that a beverage-focused lower-calorie FOP labelling strategy may be part of a comprehensive strategy to stem rising rates of obesity and NCDs.

## Supplementary information

**Additional file 1: Table S1.** Full category list, number of products, and percentage of products per category receiving the Lower Calorie Label in the Within-category and Across-category arms; **Table S2.** Group Shopping Order; **Table S3.** Regression Coefficients for Beverages Only with Moderator Mood (*N* = 184); **Table S4.** Regression Coefficients for Beverages Only with Moderator Hunger (*N* = 184).

## Data Availability

The datasets used and/or analysed during the current study are available from the corresponding author on reasonable request.

## Abbreviations

BMI: Body Mass Index
FOP: Front-of-pack
HCS: Healthier Choice Symbol
MTL: Multiple Traffic Light
NCDs: Non-communicable Diseases
NIP: Nutritional Information Panel
