# Supplementary material for: Randomized trial evaluating the effectiveness of within versus across-category front-of-package lower-calorie labelling on food demand
Source: BMC Public Health. 2020 Mar 12;20:312. doi: 10.1186/s12889-020-8434-1 (PMC7068974; doi:10.1186/s12889-020-8434-1)
Supplement: Supplementary file 1 — Additional file 1: Table S1. Full category list, number of products, and percentage of products per category receiving the Lower Calorie Label in the Within-category and Across-category arms; Table S2. Group Shopping Order; Table S3. Regression Coefficients for Beverages Only with Moderator Mood (N = 184); Table S4. Regression Coefficients for Beverages Only with Moderator Hunger (N = 184). [file 12889_2020_8434_MOESM1_ESM.docx]

**Supplemental Material**

**Supplemental Table 1**: Full category list, number of products, and percentage of products per category receiving the Lower Calorie Label in the Within-category and Across-category arms

| **Full Category List in NUSMart available during the LoCal Study** | **Number of products in Category** | **Percentage of products in Category receiving Lower Calorie Label in Within-category arm (%)** | **Percentage of products in Category receiving Lower Calorie Label in Across-category arm (%)** |
| --- | --- | --- | --- |
| Hot Beverages | 175 | 21.90 | 42.9 |
| Dairy Drinks | 233 | 20.27 | 3.4 |
| Sauces & Seasonings | 356 | 20.5 | 40.2 |
| Meals & Meal Centers | 205 | 20.0 | 0 |
| Staples | 180 | 20.3 | 1.5 |
| Processed Fish, Meat & Egg Products | 252 | 18.5 | 2.8 |
| Pre-packaged Fruit & Vegetables | 234 | 20.1 | 44.9 |
| Snacks | 313 | 20.2 | 3.5 |
| Bakery | 291 | 20.1 | 2.4 |
| Spreads | 174 | 21.3 | 5.7 |
| Chocolate Confectionery | 138 | 20.7 | 1.4 |
| Dairy | 231 | 22.2 | 14.3 |
| Sweeteners & Sugar | 76 | 21.3 | 18.4 |
| Carbonated Soft Drinks | 74 | 20.0 | 21.6 |
| Juice Drinks | 133 | 20.5 | 18.8 |
| Sports & Energy Drinks | 65 | 21.0 | 13.8 |
| Sugar & Gum Confectionery | 114 | 20.4 | 11.4 |
| Soup | 83 | 20.5 | 24.1 |
| Breakfast Cereals | 85 | 23.2 | 0 |
| Desserts & Ice Cream | 97 | 22.1 | 14.4 |
| Ready-to-Drink | 90 | 21.0 | 27.8 |
| Other Beverages | 71 | 20.0 | 39.4 |

**Supplemental Material**

**Supplemental Table 2:** Group Shopping Order

| Group Number | First shop | Second Shop | Third shop |
| --- | --- | --- | --- |
| 1 | between | within | control |
| 2 | between | control | within |
| 3 | control | within | between |
| 4 | control | between | within |
| 5 | within | between | control |
| 6 | within | control | between |

| **Supplemental Material**  **Supplemental Table 3:** Regression Coefficients for Beverages Only with Moderator Mood (N=184) | | | | | |
| --- | --- | --- | --- | --- | --- |
| Outcome | Prop. of labeled products | kCal/Dollar | Total Dollar Spent | Total kCal | kCal/Serving |
| $\alpha$ (Within-category arm relative to control for happy participants) | 0.03 | -21.11 | 0.79 | -228.10 | -2.19 |
| 95% CI | -0.13; 0.18 | -56.15; 13.92 | -3.29; 4.88 | -1054.71; 598.51 | -20.62; 16.23 |
| $\beta_{A}$ (Across-category arm relative to Within-category arm for happy participants) | -0.05 | 9.73 | -0.08 | -85.45 | 1.11 |
| 95% CI | -0.17; 0.07 | -25.29; 44.75 | -4.02; 3.86 | -949.41; 778.52 | -15.83; 18.04 |
| $\beta_{2}$ (Unhappy relative to happy participants in Within-category arm) | -0.07 | 27.58 | -2.47 | -282.65 | 8.92 |
| 95% CI | -0.29; 0.14 | -24.19; 79.34 | -9.34; 4.40 | -1724.64; 1159.34 | -23.72; 41.56 |
| $\beta_{3}$ (Unhappy in Across-category arm relative to Within-category arm) | 0.20* | -34.01 | 1.59 | 58.21 | -11.47 |
| 95% CI | 0.02; 0.37 | -77.92; 9.89 | -4.46; 7.65 | -985.94; 1102.35 | -31.64; 8.70 |
| $\beta_{2}+ \beta_{3}$ (Unhappy relative to happy participants in Across-category arm) | 0.12 | -6.44 | -0.88 | -224.40 | -2.55 |
| 95% CI | -0.08; 0.33 | -55.98; 43.11 | -7.87; 6.12 | -1700.78; 1251.90 | -22.32; 20.15 |

* ***p*** <0.05; CI stands for confidence interval

| **Supplemental Material**  **Supplemental Table 4:** Regression Coefficients for Beverages Only with Moderator Hunger (N=184) | | | | | |
| --- | --- | --- | --- | --- | --- |
| Outcome | Prop. of labeled products | kCal/Dollar | Total Dollar Spent | Total kCal | kCal/Serving |
| $\alpha$ (Within-category arm relative to Control) | -0.00 | -27.24 | 0.17 | -733.16 | 3.41 |
| 95% CI | -0.10; 0.09 | -54.29; -0.19 | -2.65; 2.98 | -1363.44; -102.89 | -17.73; 24.56 |
| $\beta_{A}$ (Across-category arm relative to Within-category arm) | 0.09 | -8.62 | 4.26 | 510.80 | -7.26 |
| 95% CI | -0.06; 0.23 | -35.25; 18.00 | 0.04; 8.47 | -190.98; 1212.58 | -23.57; 9.05 |
| $\beta_{2}$ (Hungry relative to non-hungry participants in Within-category arm) | -0.03 | 46.23 | -1.78 | 688.86 | -0.77 |
| 95% CI | -0.20; 0.15 | -4.80; 97.25 | -7.27; 3.71 | -774.39; 2152.10 | -36.74; 35.20 |
| $\beta_{3}$ (Hungry in Across-category arm relative to Within-category arm) | -0.06 | -3.77 | -6.67* | -1,118.87* | 3.52 |
| 95% CI | -0.26; 0.15 | -43.85; 36.31 | -13.33; -0.00 | -2127.82; -109.92 | -14.30; 21.34 |
| $\beta_{2}+ \beta_{3}$ (Hungry relative to non-hungry participants in Across-category arm) | -0.08 | 42.46 | -8.45* | -430 | 2.75 |
| 95% CI | -0.26; 0.09 | -8.53; 93.45 | -13.92; -2.98 | -1884.23; 1024.21 | -33.45; 38.95 |

* ***p*** <0.05; CI stands for confidence interval
